# Whole Genome Phylogeny of *Bacillus* by Feature Frequency Profiles (FFP)

**DOI:** 10.1038/srep13644

**Published:** 2015-09-01

**Authors:** Aisuo Wang, Gavin J. Ash

**Affiliations:** 1 NSW Department of Primary Industries, Wagga Wagga Agricultural Institute, PMB, Wagga Wagga, NSW, 2650, Australia; 2 Graham Centre for Agricultural Innovation, Locked bag 588, Wagga Wagga, NSW, 2678, Australia; 3 School of Agricultural and Wine Sciences, Charles Sturt University, Wagga Wagga, NSW, Australia

## Abstract

Fifty complete *Bacillus* genome sequences and associated plasmids were compared using the “feature frequency profile” (FFP) method. The resulting whole-genome phylogeny supports the placement of three *Bacillus* species (*B. thuringiensis, B. anthracis* and *B. cereus*) as a single clade. The monophyletic status of *B. anthracis* was strongly supported by the analysis. FFP proved to be more effective in inferring the phylogeny of *Bacillus* than methods based on single gene sequences [16s rRNA gene, *GryB* (gyrase subunit B) and *AroE* (shikimate-5-dehydrogenase)] analyses. The findings of FFP analysis were verified using kSNP v2 (alignment-free sequence analysis method) and Harvest suite (core genome sequence alignment method).

Members of the genus *Bacillus* comprise gram-positive, spore forming, rod-shaped, aerobic bacteria. Three species of the *Bacillus* (*Bacillus thuringiensis, Bacillus anthracis* and *Bacillus cereus*) have a huge impact on human activities. For example, *B. anthracis* is the cause of the acute and often lethal disease anthrax^1^, which is therefore of a concern as a possible agent in biological warfare; *B. thuringiensis* is extensively used in the biological control of insects due to its ability to produce parasporal protein crystals with insecticidal activity^2^; *B. cereus* is an opportunistic human pathogen involved in food-poisoning incidents and contaminations in hospitals^1^. Some strains of *B. cereus* have been developed as a useful biological control agent in the suppression of fungi and crop disease^3^.

While the phenotypes of these *Bacillus* species are different, their intra and inter phylogenetic relationships are not clear. Several approaches have been used to classify *B. thuringiensis* strains, including rRNA gene sequences^2^, amplified fragment length polymorphisms (AFLP)^2^, restriction fragment length polymorphisms (RFLPs) in small subunit (SSU) rRNA sequences^4^, *GryB* (gyrase subunit B) and *AroE* (shikimate-5-dehydrogenase) gene sequences^5^. The results of these approaches suggest that there is a high level of sequence homology among the strains of *B. thuringiensis*. Similarly, overall genetic studies have shown that *B. thuringiensis* and *B. cereus* are essentially identical^6^. *B. anthracis* can only be distinguished from *B. thuringiensis* and *B. cereus* through microbiological and biochemical tests^7^. Since these genetic methods are not able to easily distinguish different members of *B. thuringiensis, B. anthracis* and *B. cereus*, it becomes necessary to look for some more easily recognizable markers.

With the advent and development of next generation sequencing technologies, a great deal of sequencing data has been generated in recent years. The rapid accumulation of whole genome data of *Bacillus* species in Genbank makes it possible for comparisons of genomic differences over the entire genome that can’t be identified in analyses of specific single gene sequences. However, the size of the whole genome data poses great challenges on alignment-based algorithms, which are effective in dealing with closely related sequences but are unable to evaluate the recombination, shuffling, and rearrangement events of the whole genomes^8^. Thus, alignment-free sequence analysis approaches, such as FFP (Feature Frequency Profile), provide attractive alternatives over alignment-based approaches.

FFP is a new method used to study the whole genome phylogeny based on k –mers^9^,^10^,^11^. In this method, the number of features of a particular length l that occur in a particular genome is counted and assembled into a FFP vector. FFPs from different species are then compared using the Jensen–Shannon (JS) Divergence^12^. A neighbor-joining phylogenetic tree can thus be constructed based on the resulting distance matrix. Compared to the traditional multiple sequences alignment (MSA) based method, the alignment free FFP method can compare both genic and non-genic regions of the whole genome at higher speed; it can incorporate a wide variety of genomic features into each comparison including intron deletions, exon sequence indels, transposable element insertions, base transversions in coding sequences, and some rare genomic changes such as short interspersed element/long interspersed element (SINE/LINE) insertions^13^. Benefitting from these advantages, this method has been successfully applied to resolving relationships among *Escherichia coli* and *Shigella strains*^10^, prokaryotes^9^ and mammals^13^.

In this study, we reconstructed the whole-genome phylogeny of *Bacillus* (with an emphasis on *B. thuringiensis, B. anthracis* and *B. cereus*) using the FFP approach, with an aim to better understand the phylogenetic relationships that exist among them. To validate the usefulness of FFP method, we also processed the data with kSNP v2 (alignment-free sequence analysis method) and Harvest Suite (core genome sequence alignment method). For comparison purpose, we constructed phylogenetic trees inferred from three single genes: 16s rRNA genes, *GyrB* and *AroE*, whose DNA sequences were extracted from the corresponding genomes.

## Results

### The phylogenetic results based on the whole genome data

**Figure 1:**
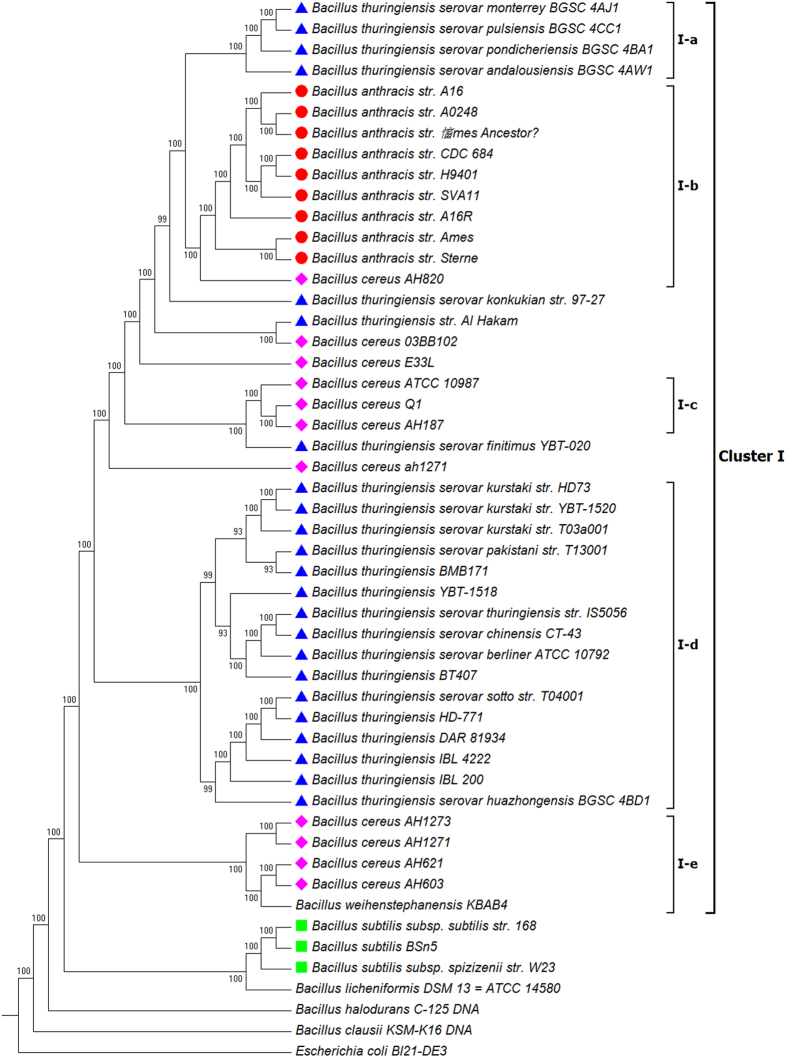
Phylogenetic tree of 50 Bacillus strains. The tree was constructed using the NJ algorithm based on the FFP features of the Whole Genome Data. *Escherichia coli* Bl21 (DE3) (AM946981.2) was used as an outgroup in the analysis. The bootstrap confidence values were generated using 1,000 permutations. Different symbols were allocated to represent different species: Blue triangle for *Bacillus thuringiensis*; Pink diamond for *Bacillus cereus*; Red circle for *Bacillus anthracis*; Green Square for *Bacillus subtilis.*

The phylogenetic tree inferred from the whole genome data of 51 taxa (Table 1) (including 23 *B. thuringiensis* strains, nine *B. anthracis* strains, 11 *B. cereus* strains, three *B. subtilis* strains, one *B. licheniformis* strain, one *B. weihenstephanensis* strain, one *B. clausii* strain, one *B. halodurans* strain and one *E. coli* strain) is presented in Fig. 1. A cluster (I) containing all the *B. thuringiensis, B. anthracis* and *B. cereus* strains apart from other *Bacillus* members under study can be recognized (with an exception of *B. weihenstephanensis*) . This cluster could be further sub-divided into at least five sub-clusters (I-a to I-e, Fig. 1). The sub-cluster I–b contains all nine *B. anthracis* strains (*B. anthracis* str. A0248, *B. anthracis* str. A16, *B. anthracis* str. A16R, *B. anthracis* str. Ames, *B. anthracis* str. ‘Ames Ancestor’, *B. anthracis* str. CDC 684, *B. anthracis* str. H9401, *B. anthracis* str. Sterne, *B. anthracis* str. SVA11), whereas the sub-cluster of I-a and I-d contain exclusively *B. thuringiensis* strains (*B. thuringiensis* BMB171, *B. thuringiensis* Bt407, *B. thuringiensis* DAR 81934, *B. thuringiensis* HD-771, *B. thuringiensis* IBL 200, *B. thuringiensis* IBL 4222, *B. thuringiensis* serovar andalousiensis BGSC 4AW1, *B. thuringiensis* serovar berliner ATCC 10792, *B. thuringiensis* serovar chinensis CT-43, *B. thuringiensis* serovar huazhongensis BGSC 4BD1, *B. thuringiensis* serovar kurstaki str. HD73, *B. thuringiensis* serovar kurstaki str. T03a001, *B. thuringiensis* serovar kurstaki str. YBT-1520, *B. thuringiensis* serovar monterrey BGSC 4AJ1, *B. thuringiensis* serovar pakistani str. T13001, *B. thuringiensis* serovar pondicheriensis BGSC 4BA1, *B. thuringiensis* serovar pulsiensis BGSC 4CC1, *B. thuringiensis* serovar sotto str. T04001, *B. thuringiensis* serovar thuringiensis str. IS5056, *B. thuringiensis* YBT-1518). Three *B. cereus* strains (*B. cereus* ATCC 10987, *B. cereus* Q1 and *B. cereus* AH187) form a monophyletic clade in sub-cluster I-c, whilst four other *B. cereus* strains (*B. cereus* AH1271, *B. cereus* AH1273, *B. cereus* AH603, *B. cereus* AH621) are closely grouped with *Bacillus weihenstephanensis KBAB4* in sub-cluster I-e.

In the topology of this NJ tree, three *B. subtilis* strains (*B. subtilis* BSn5, *B. subtilis* subsp. spizizenii str. W23, *B. subtilis* subsp. subtilis str. 168) form a monophyletic clade, which is further grouped with *B. licheniformis* DSM 13 = ATCC 14580. These *Bacillus* strains together with the remaining *Bacillus* members (*B. clausii* KSM-K16 DNA, *B. halodurans* C-125 DNA) are placed near the outgroup *E. coli* (*Escherichia coli* BL21(DE3).

### Validation of FFP results

**Figure 2:**
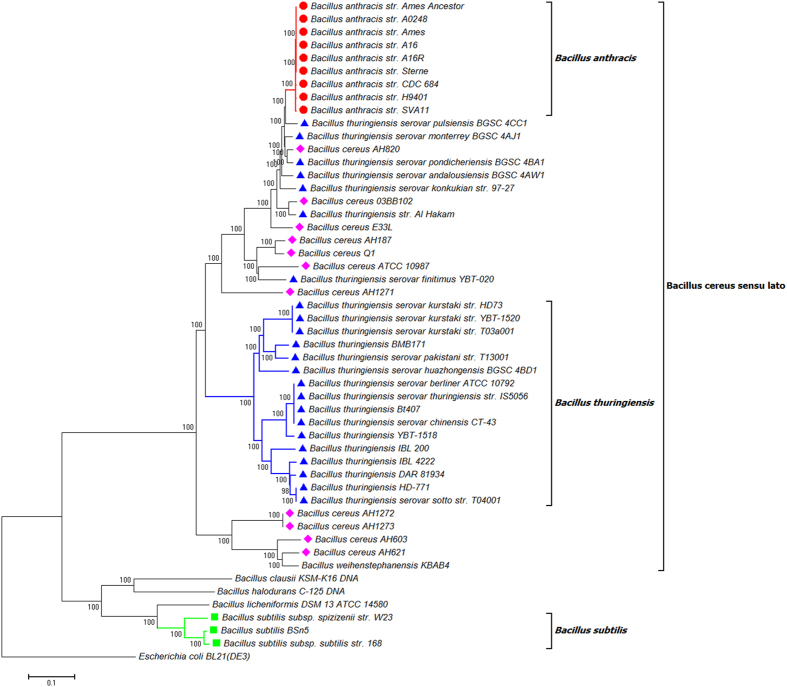
Phylogenetic tree of 50 Bacillus strains. The tree was constructed using the NJ algorithm based on all SNP matrix inferred from the kSNP V2 analysis. *Escherichia coli* Bl21 (DE3) (AM946981.2) was used as an outgroup in the analysis. The bootstrap confidence values were generated using 1,000 permutations. Different symbols were allocated to represent different species: Blue triangle for *Bacillus thuringiensis*; Pink diamond for *Bacillus cereus*; Red circle for *Bacillus anthracis*; Green Square for *Bacillus subtilis.*

The NJ tree inferred from the kSNP analyses of the whole genome is presented in Fig. 2. The monophyly of *B. anthracis* was confirmed with high bootstrap support (100). A monophyletic clade containing 16 *B. thuringiensis* isolates was recognized (clade *Bacillus thuringiensis*). All the *B. anthracis*, *B. cereus, B. thuringiensis* and *B. weihenstephanensis* form a monophyletic clade (*Bacillus cereus sensu lato*), which is separated from the remaining *Bacillus* species examined in this study. Outside this major clade, the monophyly of *B. subtilis* was confirmed (100 bootstrap support).

**Figure 3:**
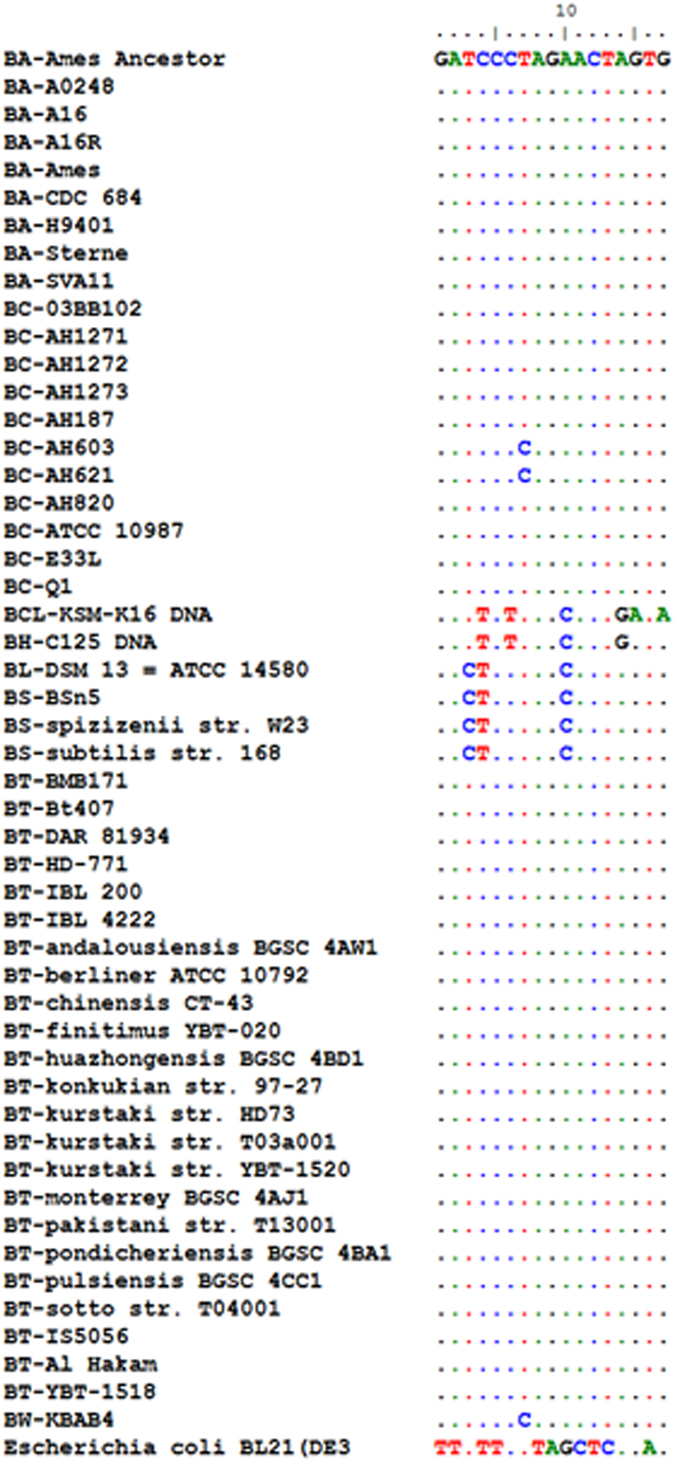
Core SNP matrix inferred from kSNP v2 (BA: *Bacillus anthracis*, BC: *Bacillus cereus*, BCL: *Bacillus clausii*, BH: *Bacillus halodurans*, BL: *Bacillus licheniformis*, BS: *Bacillus subtilis*, BT: *Bacillus thuringiensis*, BW: *Bacillus weihenstephanensis*).

The core SNP matrix resulted from the kSNP analysis provided a direct visualization of the relationships among all the *Bacillus* species studied (Fig. 3). There was no variation between the core SNPs of *B. anthracis* and *B. thuringiensis*, whist only single variation was found for two *B. cereus* strains (*Bacillus cereus AH603* and *Bacillus cereus AH621*) and *B. weihenstephanensis*. The variation of core SNP increased to two among the *B. subtilis* species and the *B. licheniformis* DSM 13 = ATCC 1458034. The sharp increase of core SNP variations in *B. halodurans* C-12533 and *B. clausii KSM-K16* (4 and 5 respectively) revealed their distant relationships to the remaining *Bacillus* species.

**Figure 4:**
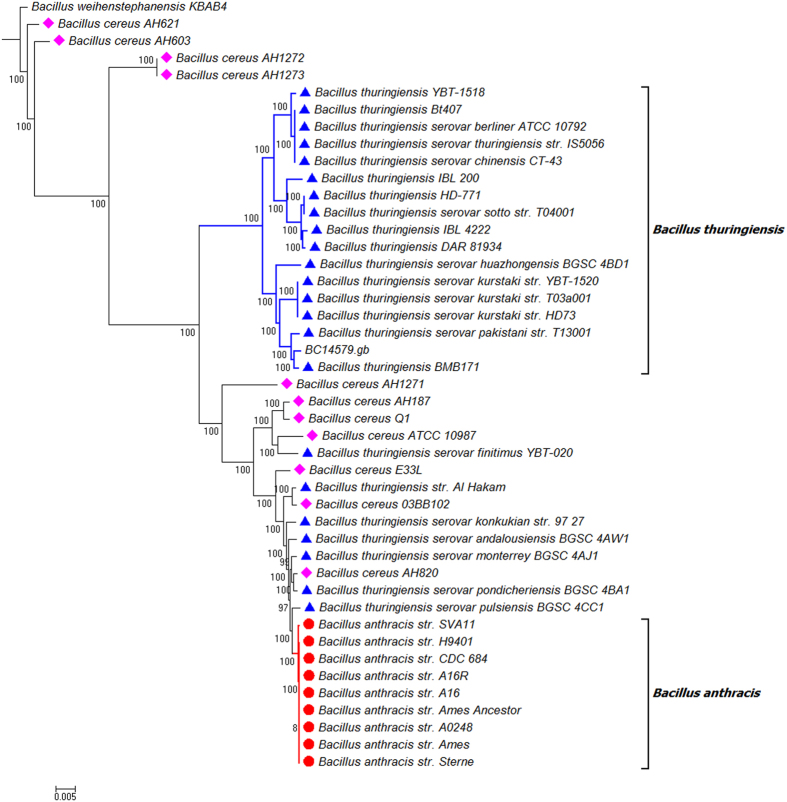
NJ tree of 44 Bacillus strains. The tree was constructed using Parsnp and annotated in MEGA 6.0. The NJ algorithm was based on the Core Genome SNP data of the 44 Bacillus strains. *Bacillus weihenstephanensis* KBAB4 was used as an outgroup in the analysis. The bootstrap confidence values were generated using 1,000 permutations. Different symbols were allocated to represent different species: Blue triangle for *Bacillus thuringiensis*; Pink diamond for *Bacillus cereus*; Red circle for *Bacillus anthracis*. The Genbank file of *Bacillus cereus* ATCC 14579 (represented as BC14579) was used as reference in the Parsnp analysis.

**Figure 5:**
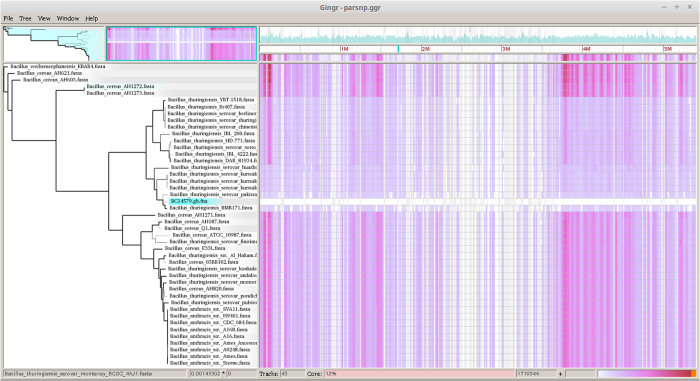
Gingr visualization of 44 Bacillus genomes aligned with Parsnp. The leaves of the reconstructed phylogenetic tree (left) are paired with their corresponding rows in the multi-alignment.

**Figure 6:**
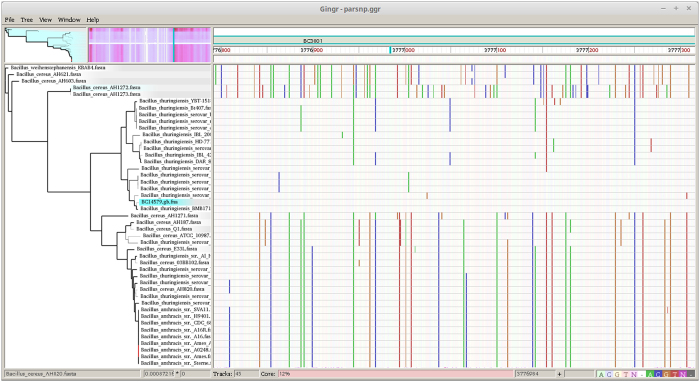
Gingr visualization of 44 Bacillus genomes aligned with Parsnp. The visual layout is the same as Fig. 5, but unlike Fig. 5, the genome alignment was zoomed to reveal the phylogenetic signature of several clades, in this case within the fully-aligned BC3801 (dipicolinate synthase subunit A).

Our effort in using Harvest suite to analyse all the species examined in FFP was not successful. The shared core genome among all the studied taxa was too low (less than 1%) to let the Parsnp program to work. This is because Parsnp is designed for intraspecific alignments and requires >=97% average nucleotide identity among input genomes. The Parsnp started to work when *Bacillus* species other than the member of *Bacillus cereus sensu lato* were excluded from the analysis. The final alignment and the resulting NJ tree are presented in Figs 4, 5, 6. The NJ tree distinguished two highly supported clades (100 in bootstrap value): one including all the *B. anthracis* strains and the other including sixteen *B. thuringiensis* strains. The whole topology of this NJ tree is highly similar to that of the NJ trees inferred from FFP and kSNP analyses (Figs 1 and 2).

The Gingr visualization of NJ tree and the genome alignment (core genome based) displayed multiple conserved regions (represented by the SNP heatmap) throughout the entire genome across 44 members of *Bacillus cereus sensu lato* (Figs 5 and 6). These conserved regions are scattered throughout the genome but showed more density in four regions (500–1500 bp; 11000–15000 bp, 36000–46000 bp and 52000–53000 bp). When being zoomed, the Gingr visualization turned the SNP heatmap into vertical lines, which revealed the phylogenetic signature of several clades [in this case within the fully—aligned dpaA gene (BC3801)] (Fig. 6).

### The phylogenetic results based on the single gene data

**Figure 7:**
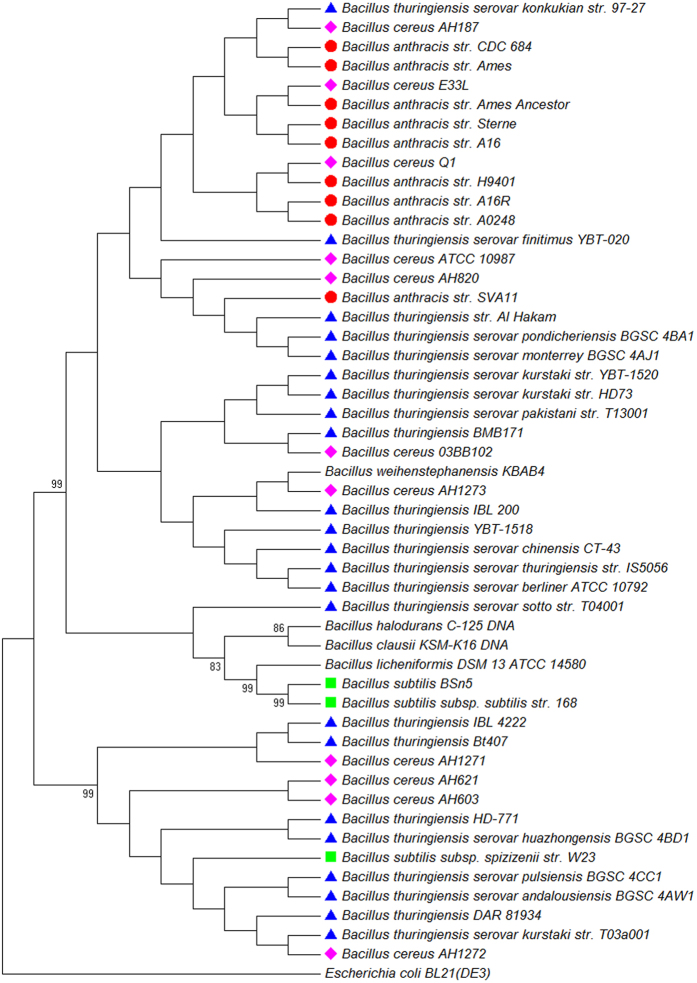
Neighbor-joining tree constructed based on the sequences of the 16S rRNA gene from 50 Bacillus strains. *Escherichia coli* Bl21 (DE3) (AM946981.2) was used as an outgroup in the analysis. The bootstrap confidence values were generated using 1,000 permutations. Different symbols were allocated to represent different species: Blue triangle for *Bacillus thuringiensis*; Pink diamond for *Bacillus cereus*; Red circle for *Bacillus anthracis*; Green Square for *Bacillus subtilis*.

**Figure 8:**
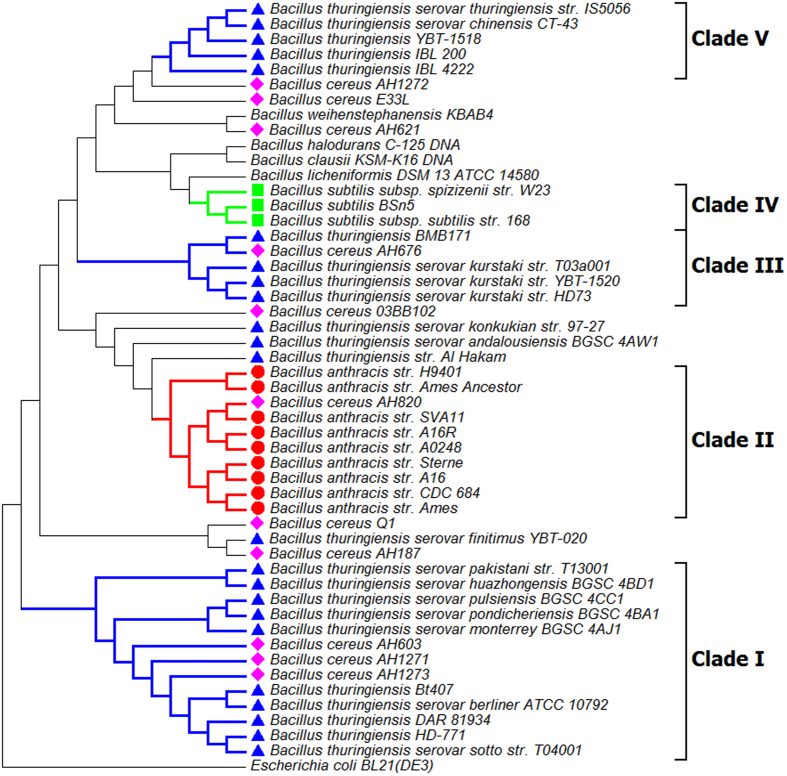
Neighbor-joining tree constructed based on the sequences of the *GyrB* from 50 Bacillus strains. *Escherichia coli* Bl21 (DE3) (AM946981.2) was used as an outgroup in the analysis. The bootstrap confidence values were generated using 1,000 permutations. Different symbols were allocated to represent different species: Blue triangle for *Bacillus thuringiensis*; Pink diamond for *Bacillus cereus*; Red circle for *Bacillus anthracis*; Green Square for *Bacillus subtilis*.

**Figure 9:**
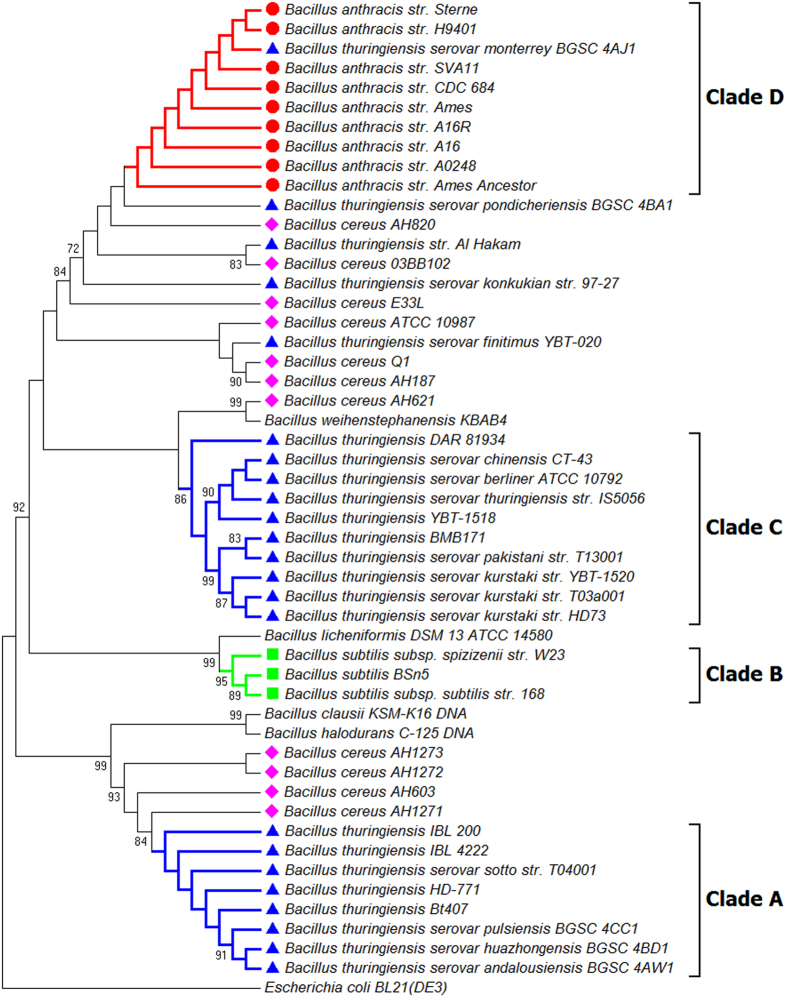
Neighbor-joining tree constructed based on the sequences of the *AroE* from 50 Bacillus strains. *Escherichia coli* Bl21 (DE3) (AM946981.2) was used as an outgroup in the analyse. The bootstrap confidence values were generated using 1,000 permutations. Different symbols were allocated to represent different species: Blue triangle for *Bacillus thuringiensis*; Pink diamond for *Bacillus cereus*; Red circle for *Bacillus anthracis*; Green Square for *Bacillus subtilis*.

Three NJ trees inferred from the data of three single genes (16s rRNA gene, *GryB* and *AroE*), are shown in Figs 7, 8, 9 respectively. These trees did not support the monophyletic status of *B. anthracis*. The clades that contain *B. anthracis* strains also contain other *Bacillus* species (e.g. *B. cereus* AH820 in Clade II of Fig. 8, and *B. thuringiensis* serovar monterrey BGSC 4AJ1 in Clade D of Fig. 9). Among the total 23 *B. thuringiensis* strains studied, some strains form monophyletic sub - clades in trees inferred from *GyrB* (Clade V, Fig. 8) and *AroE* (Clade A and C, Fig. 9), but the monophyletic status of the whole *B. thuringiensis* strains cannot be confirmed by these analyses. Similarly, *B. cereus* proved to be a paraphyletic group by all NJ trees inferred from data of three single genes. The data for *GyrB* and *AroE* suggested that *B. subtilis* might be a monophyletic group (Clade IV in Fig. 8 and Clade B in Fig. 9), and this group has close relationship with *B. licheniformis* DSM 13 ATCC 14580, which is supported by high bootstrap value (97 in Fig. 8 and 99 in Fig. 9). With respect to the phylogenetic placement of *B. subtilis* and *B. licheniformis*, the 16S rRNA gene shows very low support in comparison to the other two protein coding genes (Fig. 7).

## Discussion

**Figure 10:**
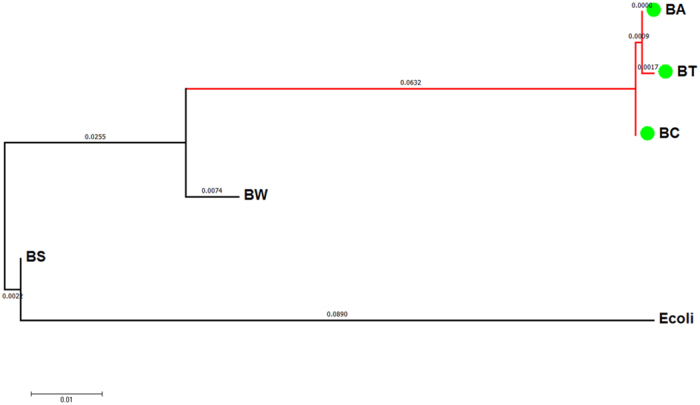
Pairwise Distance of the Bacillus numbers. The scale bar represents a 1% sequence difference (BA:*B. anthracis*; BT:*B. thuringiensis*; BC:*B. cereus*; BW:*B. weihenstephanensis*; BS:*B. subtilis* and *B. clausii*, *B. halodurans*, *B. licheniformis*) are placed near the outgoup *E. coli* (*Escherichia coli* BL21(DE3).

Our phylogenetic analysis based on the FFP features of the whole genome and associated plasmids resulted in a major cluster containing all strains of *B. thuringiensis, B. anthracis* and *B. cereus* separated from other recognised *Bacillus* members. When strains of same species were grouped together and subject to pairwise distance analysis, the groups of *B. thuringiensis, B. anthracis* and *B. cereus* formed a monophyletic clade in the NJ tree (Fig. 10). These results clearly suggest the close relationship among *B. thuringiensis, B. anthracis* and *B. cereus* species, and are in agreement with earlier results from DNA-DNA hybridization analysis and Multi Locus Enzyme Electrophoresis (MEE), which showed high identity among *B. anthracis*, *B. cereus*, and *B. thuringiensis* strains^14^. These three species have been grouped under the name of *Bacillus cereus sensu lato*^15^ despite their obvious difference in phenotype and pathological effects, which are resulted from the genetic difference in plasmid rather than in chromosome^1^. The results of present study appear to support the classification of *Bacillus cereus sensu lato* when using genomic sequences only (data not shown). Greater resolution of recognised species was achieved when plasmid sequences were added to the analysis.

In the present study, *B. weihenstephanensis* strain KBAB4 was found to be very closely grouped with the major cluster I-d consisting of all *B. thuringiensis* isolates and proximal to cluster I-e (*B. cereus*) and cluster I-c (a cluster containing both *B. thuringiensis* and *B. cereus* strains) (Fig. 1). *B. weihenstephanensis* is a member of the *Bacillus cereus sensu lato*, and has high similarities with *B. thuringiensis* and *B. cereus* in terms of its ecological features such as producing cereulide as *B. cereus* and being psychrotolerance as some *B. thuringiensis* isolates^16^,^17^. Soufiane and Cote (2009)^5^ revealed the close relationship between *B. weihenstephanensis* and some *B. thuringiensis* serovars based on the 16S rRNA, *GyrB* and *AroE* gene sequences. Our results support their research and provide further evidence for the classification of *Bacillus cereus sensu lato*.

The NJ tree inferred from the whole genome sequences of these bacteria species not only revealed the close relationship among *B. thuringiensis, B. anthracis* and *B. cereus*, but also confirmed the monophyly of *B. anthracis* (I-b, Fig. 1). Previous studies using other techniques have all stated that *B. anthracis* is the most monomorphic species among *B. thuringiensis, B. anthracis* and *B. cereus*^18^,^19^,^20^. Our results confirmed the genetic homogeneity of *B. anthracis* but failed to elicidate the evolutionary relationships between *B. anthracis* and the remaining two species. *B. anthracis* has been regarded to be evolved from a *B. cereus* ancestor through acquisition of key plasmid-encoded toxin, capsule and regulatory loci^21^. Such a relationship did not appear in our phylogenetic analyses based on FFP analysis of whole genome data (Fig. 1). The *B. anthracis* clade is proximal to a number of isolates of *B. cereus* and *B. thuringiensis* which have been associated with disease or toxicity in humans.

The findings of FFP analyses were fully supported by SNP phylogenies construed by kSNP (alignment – free sequence analysis method) and Parsnp (core genome alignment method). By comparing the NJ trees inferred from FFP analysis (Fig. 1) and kSNP analysis (Fig. 2), we found a high level of similarity between two phylogenies. The clades of I - b and I - d clades in FFP tree are consistent with the *Bacillus anthracis* and *Bacillus thuringiensis* clades in kSNP tree, whilst the cluster I in FFP tree is corresponding to the clade of *Bacillus cereus sensu lato* in kSNP tree. While the core genome SNP tree constructed by Parsnp failed to cover all the species studied, the exclusion of other *Bacillus* species from the major cluster was actually a support for the monophyly of *Bacillus cereus sensu lato*. This is because Parsnp is limited in intraspecific alignment and can only tolerate genomes with high similarity ( >=97%). Genomes from other species will be automatically excluded from the full alignment^22^.

Within the core genome SNP tree constructed by Parsnp and visualized by Gingr, the monophyly of *B. anthracis* and a subclade covering 16 *B. thuringiensis* strains were confirmed, which is consistent with the results of FFP analysis. The tree also revealed the paraphyly of *B. cereus* and *B. thuringiensis*, which is similar to the findings of FFP and kSNP analyses. By zooming the alignment files from genome level to nucleotide level via the fisheye zoom feature of Gingr^22^, we noticed the SNP variations across different strains of *B. cereus* and *B. thuringiensis* that affects the topology of the trees. For *B. cereus*, the most variable region falls on an area between the gene of *Cytochrome d ubiquinol oxidase subunit II* and the gene of *Alanine racemase* (around 121456 bp). There are more SNP sites at this region among four *B. cereus* strains (*B. cereus AH603, B. cereus AH621, B. cereus AH1272* and *B. cereus AH1273*), which contributed the distant placement of these four strains from the remaining *B. cereus* strains in the NJ tree. Similarly, we found a region (around 1006988 bp) with high SNP density in *B. thuringiensis* (starting from the gene of *Lysr-type transcriptional regulator* and ending at the gene of *Thiamine/molybdopterin biosynthesis protein*). The distantly placed *B. thuringiensis* strains (such as *B. thuringiensis* serovar *finitimus YBT-020* and *B. thuringiensis str. Al Hakam*) generally have more SNP sites in this region than that of the remaining *B. thuringiensis* strains. It is not clear why some *B. cereus* and *B. thuringiensis* strains have more SNP variations at these particular genome regions than that of other strains, and what are the impacts of these SNP variations on the phenotype, function and pathogenicity of these *Bacillus* strains. More research is thus required to answer these questions.

In contrast to the FFP analysis based on the whole genome data, our phylogenetic analysis based on single gene data (16S rRNA gene, *GyrB* and *AroE*) were unable to clearly distinguish between *Bacillus* species examined. The 16S rRNA gene sequence analysis clustered all the sequences together and provided poor resolution for the relationships between each strain. Similarly, our analysis based on two protein coding genes, *GyrB* and *AroE*, were unable to separate *B. thuringiensis, B. anthracis* and *B. cereus* from other *Bacillus* members while they provided support for the monophyletic position of *B. subtilis*. A further analysis using the concatenated sequence of these genes failed to provide any better analysis (data not shown).

These results suggest that FFP analysis of the combined genomic and plasmid sequence data allows for comparisons of genomic differences that can’t be identified in analyses of specific single gene sequences and provides greater resolution of species belonging to *B. cereus senso lato* than other techniques such as MLST, AFLP or single gene sequence analysis. Furthermore, the availability and reduced cost of whole genome sequencing can be used without extensive gene annotation to provide robust phylogenetic analysis of new isolates as they become available.

## Materials and Methods

### Source of sequence data

The genome sequence of *Bacillus thuringiensis* strain DAR 81934 was from our previous research^23^. Genome sequences of other 49 *Bacillus* and one *E.coli* [*Escherichia coli* BL21(DE3)] (used as outgroup) were retrieved from GenBank (Table 1). The retrieved genome sequences cover both the main chromosome sequences and the plasmid sequences (if any) of each species. The nucleotide sequences of three single nuclear genes for these taxa: 16s rRNA, *GyrB* and *AroE*, were extracted from the corresponding whole genome sequences.

### Phylogeny analysis via FFP

The whole genome sequences of the 51 taxa were converted to multiFasta format before being uploaded to FFP –3.19^10^, where the different forms of genome partitions were compared between species, and NJ trees were constructed based on the Jensen–Shannon divergences matrix from each type of genome partition. By following the recommendations of the program, we used the tools of ffpvocab and ffpre to find the right range of lengths to use (l = 20 was finally chosen in the analysis). We also conducted bootstrapping (1000) to assess the FFP phylogenetic analysis. The outcome of the bootstrap analysis was imported into Phylip 3.2^24^ to create a consensus distance matrix, which was further processed in MEGA 6.0^25^ to display the final NJ tree.

### Validation of FFP results

We applied two programs to validate the outcomes of FFP analysis. The first program is kSNP v2.1.2^26^, an alignment-free sequence analysis method with the capacity to build whole genome phylogeny on single nucleotide polymorphisms (SNPs) in whole genome data. We examined the same datasets of FFP by running kchooser to find the optimum k –mer size (19) prior to the kSNP analysis, and including the flag of “–j ” in the command line to estimate Neighbor Joining trees based on all SNPs and core SNPs. The resulting all-SNPs-matrix was imported into MEGA 6.0^25^ for NJ tree construction. The core-SNP-smatrix was applied to demonstrate the core SNP differneces across all examined genomes.

The Harvest Suite^22^ (inclusing Parsnp and Gingr) was also applied to validate the FFP outcome. We aligned genomes studied in FFP and built NJ phylogentic trees through Parsnp, a fast core-genome multi-aligner, and vusualized the alignment and trees with Gingr, a dynamic visual platform. The default parameters recommanded by the program were followed during the whole analysis.

### Single gene based phylogeny

The retrieved single gene sequences of 16s rRNA, *GyrB* and *AroE* were imported into MEGA 6.0 for sequence alignment (Clustal W^27^) and phylogenetic analysis (Neighbor-Joining^28^). The Kimura 2-parameter model was selected by executing the function of “Find Best DNA/Protein Models” prior to the Phylogenetic analyses. Statistical confidence on the inferred tree topology was assessed with 1,000 bootstrap replications.

## Additional Information

**How to cite this article**: Wang, A. and Ash, G. J. Whole Genome Phylogeny of *Bacillus* by Feature Frequency Profiles (FFP). *Sci. Rep.* **5**, 13644; doi: 10.1038/srep13644 (2015).

## Figures and Tables

**Table 1:** Source of sequence data.

**Species**	**Assess No.**	**Genome size (bp)**	**Plasmid (Accession No., Size in bp)**
*Bacillus anthracis str. A0248*	CP001598.1	5227419	Pxo1(CP001599.1, 181677); Pxo2(CP001597.1, 94830)
*Bacillus anthracis str. A16*	CP001970.1	5227898	pXO1(CP001971.1, 181764); pXO2(CP001972.1, 94839)
*Bacillus anthracis str. A16R*	CP001974.1	5227683	pXO1(CP001975.1, 181763)
*Bacillus anthracis str. Ames*^21^	AE016879.1	5227293	Nil
*Bacillus anthracis str. ‘Ames Ancestor*^29^	AE017334.2	5227419	pXO1(AE017336.2, 181677); pXO2(AE017335.3, 94830)
*Bacillus anthracis str. CDC 684*	CP001215.1	5230115	pX01(CP001216.1, 181773); pX02(CP001214.1, 94875)
*Bacillus anthracis str. H9401*^30^	CP002091.1	5218947	BAP1(CP002092.1, 181700); BAP2(CP002093.1, 94824)
*Bacillus anthracis str. Sterne*	AE017225.1	5228663	Nil
*Bacillus anthracis str. SVA11*^31^	CP006742.1	5210966	Pxo1(CP006743.1, 181793); pXO2(CP006744.1, 94758)
*Bacillus cereus 03BB102*	CP001407.1	5269628	p03BB102_179(CP001406.1, 179680)
*Bacillus cereus AH1271*	CM000739.1	5656704	Nil
*Bacillus cereus AH1272*	CM000740.1	5789540	Nil
*Bacillus cereus AH1273*	CM000741.1	5790501	Nil
*Bacillus cereus AH187*	CP001177.1	5269030	pAH187_12(CP001178.1, 12481); pAH187_270(CP001179.1, 270082); pAH187_3(CP001181.1, 3091); pAH187_45(CP001180.1, 45173)
*Bacillus cereus AH603*	CM000737.1	5799451	Nil
*Bacillus cereus AH621*	CM000719.1	5674808	Nil
*Bacillus cereus AH820*	CP001283.1	5302683	pAH820_10(CP001286.1, 10915); pAH820_272(CP001285.1, 272145); pAH820_3(CP001284.1, 3091)
*Bacillus cereus ATCC 10987*^32^	AE017194.1	5224283	pBc10987(AE017195.1, 208369)
*Bacillus cereus E33L*	CP000001.1	5300915	pE33L466(CP000040.1, 466370); pE33L5(CP000041.1, 5108); pE33L54(CP000042.1, 53501); pE33L8(CP000043.1, 8191); pE33L9(CP000044.1, 9150)
*Bacillus cereus Q1*^33^	CP000227.1	5214195	pBc239(CP000228.1, 239246); pBc53(CP000229.1, 52766)
*Bacillus clausii KSM-K16*^34^	AP006627.1	4303871	Nil
*Bacillus halodurans C-125*^35^	BA000004.3	4202352	Nil
*Bacillus licheniformis DSM 13* = *ATCC 14580*^36^	AE017333.1	4222645	Nil
*Bacillus subtilis BSn5*^37^	CP002468.1	4093599	Nil
*Bacillus subtilis subsp. spizizenii str. W23*^38^	CP002183.1	4027676	Nil
*Bacillus subtilis subsp. subtilis str. 168*^39^,^40^,^41^	CM000487.1	4214547	Nil
*Bacillus thuringiensis BMB171*^42^	CP001903.1	5643051	pBMB171(CP001904.1, 312963)
*Bacillus thuringiensis Bt407*^43^	CM000747.1	6026843	BTB_15p(CP003892.1, 15189); BTB_2p(CP003897.1, 2062); BTB_502p(CP003890.1, 501911); BTB_5p(CP003896.1, 5518); BTB_6p(CP003895.1, 6880); BTB_78p(CP003891.1, 77895); BTB_7p(CP003894.1, 7635); BTB_9p(CP003898.1, 8513)BTB_8p(CP003893.1, 8240);
*Bacillus thuringiensis DAR 81934*^23^	CM001804.1	5628425	Nil
*Bacillus thuringiensis HD-771*	CP003752.1	5883036	p01(CP003753.1, 171030); p02(CP003754.1, 168999); p03(CP003755.1, 69876); p04(CP003756.1, 65470); p05(CP003757.1, 45262); p06(CP003758.1, 14056); p07(CP003759.1, 9070); p08(CP003760.1, 8574)
*Bacillus thuringiensis IBL 200*	CM000758.1	6731790	Nil
*Bacillus thuringiensis IBL 4222*	CM000759.1	6616432	Nil
*Bacillus thuringiensis serovar andalousiensis BGSC 4AW1*	CM000754.1	5488844	Nil
*Bacillus thuringiensis serovar berliner ATCC 10792*	CM000753.1	6260142	Nil
*Bacillus thuringiensis serovar chinensis CT-43*^44^	CP001907.1	5486830	pCT127(CP001908.1, 127885); pCT14(CP001909.1, 14860); pCT281(CP001910.1, 281231); pCT51(CP001911.1, 51488); pCT6880(CP001912.1, 6880); pCT72(CP001913.1, 72074); pCT8252(CP001914.1, 8252); pCT83(CP001915.1, 83590); pCT8513(CP001916.1, 8513); pCT9547(CP001917.1, 9547)
*Bacillus thuringiensis serovar finitimus YBT-020*^45^	CP002508.1	5235490	pBMB26(CP002509.1, 187880); pBMB28(CP002510.1, 139013)
*Bacillus thuringiensis serovar huazhongensis BGSC 4BD1*	CM000756.1	6231196	Nil
*Bacillus thuringiensis serovar konkukian str. 97-27*	AE017355.1	5237682	pBT9727(CP000047.1, 77112)
*Bacillus thuringiensis serovar kurstaki str. HD73*^46^	CP004069.1	5646799	pAW63(CP004072.1, 71777); pHT11(CP004073.1, 11769); pHT7(CP004076.1, 7635); pHT73(CP004070.1, 77351); pHT77(CP004071.1, 76490); pHT8_1(CP004074.1, 8513); pHT8_2(CP004075.1, 8241)
*Bacillus thuringiensis serovar kurstaki str. T03a001*	CM000751.1	5527568	Nil
*Bacillus thuringiensis serovar kurstaki str. YBT-1520*	CP004858.1	5602265	pBMB11(CP004863.1, 11769); pBMB2062(CP004859.1, 2062); pBMB293(CP004861.1, 293574); pBMB422(CP004860.1, 422692); pBMB53(CP004862.1, 53838); pBMB67(CP004869.1, 67159); pBMB7635(CP004867.1, 7635); pBMB7921(CP004866.1, 7921); pBMB8240(CP004865.1, 8240); pBMB8513(CP004864.1, 8513); pBMB94(CP004868.1, 94568)
*Bacillus thuringiensis serovar monterrey BGSC 4AJ1*	CM000752.1	6489024	Nil
*Bacillus thuringiensis serovar pakistani str. T13001*	CM000750.1	6037513	Nil
*Bacillus thuringiensis serovar pondicheriensis BGSC 4BA1*	CM000755.1	6031475	Nil
*Bacillus thuringiensis serovar pulsiensis BGSC 4CC1*	CM000757.1	6002603	Nil
*Bacillus thuringiensis serovar sotto str. T04001*	CM000749.1	6107746	Nil
*Bacillus thuringiensis serovar thuringiensis str. IS5056*^47^	CP004123.1	5491935	pIS56-107(CP004134.1, 107431); pIS56-11(CP004127.1, 11331); pIS56-15(CP004128.1, 15185); pIS56-16(CP004129.1, 16206); pIS56-233(CP004135.1, 233730); pIS56-285(CP004136.1, 285459); pIS56-328(CP004137.1, 328151); pIS56-39(CP004130.1, 39749); pIS56-6(CP004124.1, 6880); pIS56-63(CP004131.1, 63864); pIS56-68(CP004132.1, 68616); pIS56-8(CP004125.1, 8251); pIS56-85(CP004133.1, 85134); pIS56-9(CP004126.1, 9671)
*Bacillus thuringiensis str. Al Hakam*^48^	CP000485.1	5257091	pALH1(CP000486.1, 55939)
*Bacillus thuringiensis YBT-1518*	CP005935.1	6002284	pBMB0229(CP005936.1, 45206); pBMB0230(CP005937.1, 49195); pBMB0231(CP005938.1, 146276); pBMB0232(CP005939.1, 171593); pBMB0233(CP005940.1, 240661)
*Bacillus weihenstephanensis KBAB4*^49^	CP000903.1	5262775	pBWB401(CP000904.1, 417054); pBWB402(CP000905.1, 75107); pBWB403(CP000906.1, 64977); pBWB404(CP000907.1, 52830)
*Escherichia coli BL21(DE3)*	AM946981.2	4558947	Nil
